# An unusual presentation of an oral human papillomavirus (HPV) lesion

**DOI:** 10.11604/pamj.2020.37.332.26648

**Published:** 2020-12-10

**Authors:** Cinzia Casu, Antonello Mameli

**Affiliations:** 1 Department of Surgical Science, Oral Biotechnology Laboratory (OBL), University of Cagliari, Cagliari, Italy

**Keywords:** Oral HPV lesion, squamous papilloma, oral HPV, lichen planus

## Image in medicine

A 45-year-old male patient went to our observation for a white lesion in the left lower gum. At the anamnesis, he reported previous diagnosis of oral erosive and genital lichen planus (discovered 2 and 12 years before respectively) and the excision of 2 small oral squamous papillomas 2 year ago. A PCR-real time test using a saliva sample was carried out a year ago, but it did not confirm the presence of the HPV DNA. During the clinical examination, we observed a white not scrapable lesion of 2.2cm in the vestibular gum around the teeth 3.6-3.7. The lesion is not linked with a traumatic event and we decided to make an incisional biopsy to verify if it was another injury linked to the spread of oral erosive lichen planus or a new entity. The histological examination confirmed a diagnosis of oral HPV lesion, precisely squamous papilloma, but in-situ hybridation did not find HPV DNA. The presence of a mild dysplasia with unaffected margins was also confirmed. At our first knowledge, this is the first documented case in literature of an oral squamous papilloma with a keratin-like appearance, rather than exophytic and/or cauliflower form. The differential diagnosis could be with oral lichen planus, leukoplakia or verruca vulgaris. The patient was advised of the risk of malignancy connected with the simultaneous presence of oral erosive lichen planus and HPV lesions.

**Figure 1:**
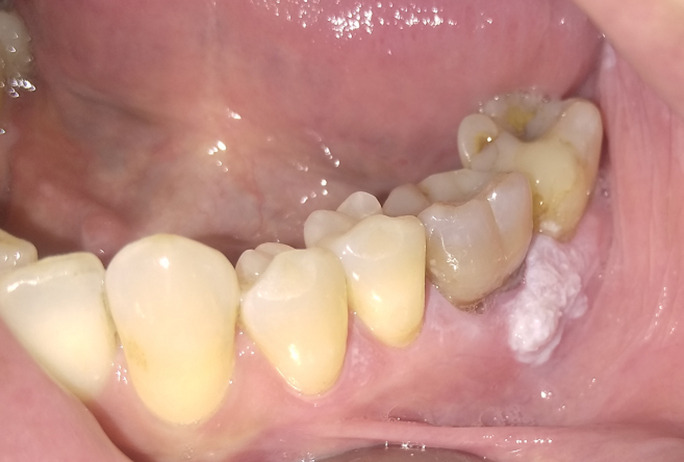
particular presentation of a relapse of squamous papilloma

